# The role of social medicine in the COVID-19 pandemic era

**DOI:** 10.7189/jogh.11.03068

**Published:** 2021-04-17

**Authors:** Luciano Bubbico, Saverio Bellizzi, Salvatore Ferlito, Luca Cegolon

**Affiliations:** 1 Department of Sensorineural Disabilities, INAPP/Italian Institute of Social Medicine, Rome, Italy; 2 Medical Epidemiologist, Independent Consultant, Geneva, Switzerland; 3 University of Catania School of Medicine, Department of Surgical Medical Sciences and Advanced Technologies, Catania, Italy; 4 Local Health Unit N.2 “Marca Trevigiana”, Public Health Department, Treviso, Italy

COVID-19 has proved to be a social disease due to its widespread diffusion in the general population, the serious harm it causes on affected patients and its impact on the economy and social life of burdened countries. While socialization is a risk factor for the spread of the SARS-C0V-2, health protection measures such as isolation and lockdown further aggravated the “social” burden of COVID-19. Diseases with social impact require a management approach based on social medicine, integrating health, social and economic responses.

There is evidence that a low socio-economic status (SES) is strongly associated with higher rates of both incidence and mortality attributable to COVID-19 [1]. In particular, housing conditions, over-crowding and other aspects that hinder social distancing can greatly influence the risk of COVID-19 transmission. Furthermore, individuals of lower SES are more likely to rely on public transport to reach their respective workplace, thereby increasing the risk of COVID-19 through inter-personal contact.

SES also affects the living environment, the eating habits, the occupational status and the access to health care services, ultimately influencing health [2]. Most determinants of health are social by nature and the most effective public health interventions to tackle them frequently require a social component in their design and implementation [3].

Given the current scenario caused by the COVID-19 pandemic, interventions to tackle socio-economic disparities should be considered as priorities like the search for effective curative and preventative treatments (eg, antivirals and vaccines). Indeed, existing inequities worsened during the pandemic and aspects like access to food, education, and psycho-social support must be carefully weighed in a holistic approach.

The application of social medicine in the current COVID-19 pandemic requires the recall of a long standing medical tradition. The history of recent epidemics, plagueing Italy in the early twentieth century, has shown that the ravages of war, movement of armies, migration of entire populations, poverty, overcrowding and poor hygienic housing conditions contributed to increase the spread of deadly communicable diseases. Social medicine, established in Italy in 1922 to contain the massive spread of tuberculosis and malaria, assumed a key role to promote the first European policies of capillary health education across Europe, contributing to prevent and control relevant infectious threats [4].

As clarified by John A. Ryle, “*social medicine extends the interest and alters the emphasis of the older public health, just as social pathology extends the interest and alters the emphasis of earlier epidemiological study”* [5]. Specifically, social medicine unites the clinical with the public and embraces the organisation of aftercare, and the readjustment of the lives of individuals and families disturbed or broken by illness.

When confronted with public health, which is primarily intended to focus on the environment (housing, safe water, and sanitation), social medicine differs by encompassing *“the whole of the economic, nutritional, occupational, educational, and psychological opportunity or experience of the individual or the community”* [5]. Basically, social medicine is concerned over the relation between the individual and his environment.

People are simultaneously biological and social organisms, and thus human health and disease are affected by social factors as well as by biological factors. Included in the basic idea and concept of social medicine is that the interdisciplinary program between medicine and social science would provide the former with knowledge and skills needed to analyze the social causes of health and illness in the same way as the alliance between medicine and laboratory sciences had provided new insights into the biological, chemical and physical bases of disease [6].

**Figure:**
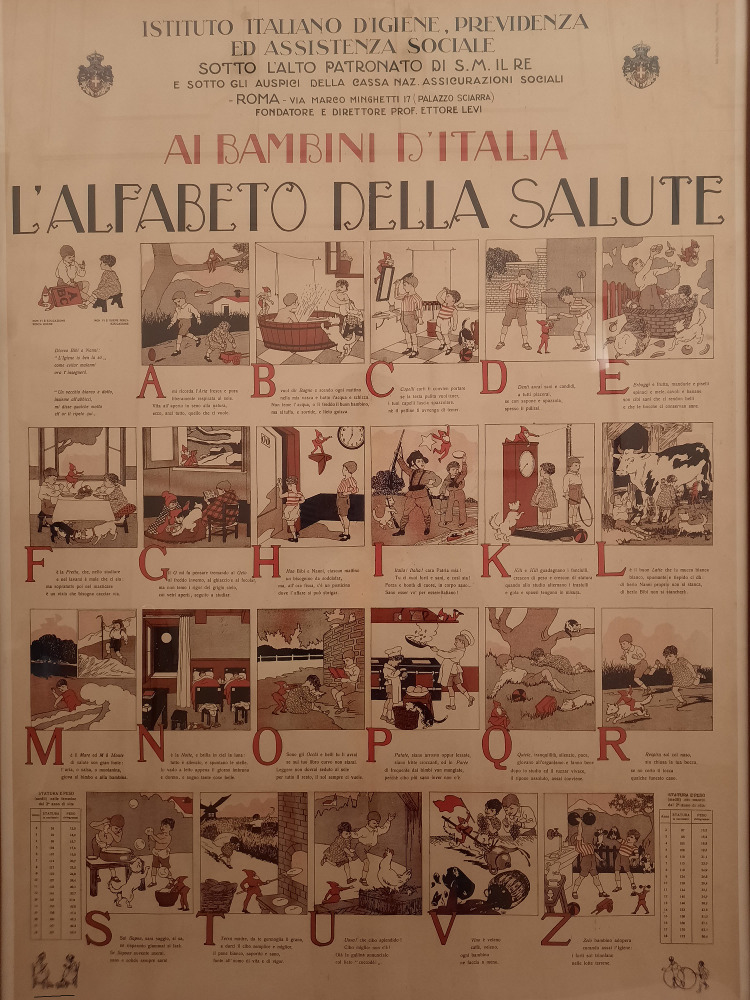
Photo: The Health Alphabet (Historical Manifesto of the Italian Institute of Social Medicie, year 1930). Prevention of epidemic diseases by health and behavioural education: each alphabetic letter corresponds to a hygiene and preventative rule. “A” like fresh and pure outdoor air (courtesy of Mrs. Arianna Cessari, used with permission).

Social medicine explicitly investigates social determinants of health and disease, rather than treating such determinants as mere background to biomedical phenomena.

The proposition of social medicine deserves emphasis, and especially so today – its intellectual breadth, its political and economic depth, its essential humanism.

Contemporary social medicine is critical to understand and prevent diseases, improving healthy life conditions in the general population and the efficiency of health systems.

Today's health challenges require novel approaches involving the implementation of new technologies and advanced methodologies such as tele-medicine, tele-rehabilitation, tele-consultation and new digital infrastructures for modern data communication 5G technology, Big Data and their management through artificial intelligence algorithms will define an epochal change in health care delivery, processing huge amounts of health data in real time [7].

These technologies will allow clinical research to identify new models of diagnostic, therapeutic and preventative interventions, optimizing health care expenditures yet supporting the most fragile population. Recent studies suggest that countries implementing an integrated health, social and economic public policy response will be able to overtake the current pandemic not only healthier, but also economically and socially stronger [8].
